# Does knowledge of liver fibrosis affect high-risk drinking behaviour (KLIFAD): an open-label pragmatic feasibility randomised controlled trial

**DOI:** 10.1016/j.eclinm.2023.102069

**Published:** 2023-06-30

**Authors:** Mohsan Subhani, Doyo G. Enki, Holly Knight, Katy A. Jones, Kirsty Sprange, Stefan Rennick-Egglestone, Joanne R. Morling, Andrew Wragg, Clare Hutton, Stephen D. Ryder

**Affiliations:** a Nottingham Digestive Diseases Centre (NDDC), School of Medicine, University of Nottingham, Nottingham, UK; b NIHR Nottingham Biomedical Research Centre, Nottingham University Hospitals NHS Trust and the University of Nottingham, Nottingham, UK; c School of Medicine, University of Nottingham, Nottingham, UK; d Nottingham Centre for Public Health and Epidemiology, University of Nottingham, Nottingham, UK; e Academic Unit of Mental Health and Clinical Neuroscience, School of Medicine, Institute of Mental Health, University of Nottingham, Nottingham, UK; f Nottingham Clinical Trials Unit, University of Nottingham, Nottingham, UK; g School of Health Sciences, Institute of Mental Health, University of Nottingham, Nottingham, UK; h Patient and Public Involvement (PPI) Co-applicant, Nottingham, UK

**Keywords:** Alcohol, Transient elastography, Alcohol-related liver disease, Alcohol recovery stories, Alcohol recovery narratives, Fibroscan, Alcohol misuse, Feasibility, RCT

## Abstract

**Background:**

Early identification followed by effective behaviour interventions is pivotal to changing the natural history of alcohol-related liver disease. We examined the feasibility of using transient elastography based advice and alcohol recovery video stories (ARVS) to change drinking behaviour in community alcohol services.

**Methods:**

A feasibility randomised control trial (RCT) was conducted in three community alcohol services. Adults 18+ years presenting with a primary alcohol problem were randomised (1:1) to receive either usual care (control group) or usual care and the KLIFAD Intervention, consisting of advice tailored to liver stiffness measure and access to ARVS (intervention group). Data were collected at baseline and six months. To establish definitive trial feasibility, recruitment and retention rates, study procedure safety and extent of effectiveness were measured (Start date: 02.10.2019, End date: 30.11.2022, ISRCTN.com: 16922410).

**Findings:**

382 service users were screened, 184 were randomised (intervention: 93, control: 91), and baseline data were collected for 128 (intervention: 71, control: 59). Six months follow-up data were available in 87 (intervention: 53, control: 34). Intervention compared to the control group had a longer duration of engagement with services (mean difference 8.6 days SD = 18.4), was more likely to complete the allocated treatment program and reduced or stop drinking (54.9% vs 43.9%) and reduce AUDIT category (71.7% vs 61.8%). There were no reported serious adverse reactions, one intervention group participant reported an increase in AUDIT category.

**Interpretation:**

Integration of transient elastography in community alcohol services is feasible. It may improve engagement with services, retention in clinical trials and supplement the reduction in self-reported alcohol consumption. A definitive RCT is supported.

**Funding:**

10.13039/501100000272National Institute for Health and Care Research (NIHR201146).

**Research in context**

**Evidence before this study**

We conducted a systematic review with a meta-analysis to review the existing literature evaluating the effectiveness of advice based on biomarkers of liver injury, including non-invasive tests for liver disease, in changing high-risk drinking behaviour and alcohol-related adverse outcomes. We searched Ovid Medline, PubMed, EMBASE, Psychinfo and CINAHL up to the end of February 2020. An additional search for grey literature was conducted using Scopus, Ethos, and Clinical Trials. A total of 14 randomised control trials and two observational studies comprising n = 3763 participants were included. The results demonstrated that advice based on biomarkers of liver injury can be effective in reducing alcohol-related harm and changing drinking behaviour. However, there was limited evidence on the effectiveness of such type of advice delivered in community alcohol services.

**Added value of this study**

To our knowledge, this is the first RCT to demonstrate that the integration of non-invasive testing of liver stiffness by transient elastography into community alcohol services is feasible. It improves the engagement and retention within community alcohol services, may supplement a change in high-risk drinking behaviour and increase trial-specific follow-up rates. It can enhance the detection rate of otherwise asymptomatic but clinically significant liver disease and can assist behaviour change. The finding of normal or elevated liver stiffness did not lead to unintended negative effects such as an exacerbation in alcohol consumption.

**Implications of all the available evidence**

The burden of undiagnosed but clinically significant liver disease, especially in a high-risk population such as those attending community alcohol services is an ongoing concern in the hepatology community. Alcohol treatment services are an ideal setting for early diagnosis of alcohol-related liver disease and interventions to prevent further physical harm. Integrating non-invasive liver stiffness testing into these services creates an opportunity to change the natural history of liver disease progression in high-risk individuals. National Institute for Health and Care Excellence guidelines state adults with high levels of alcohol dependency should be assessed and offered intensive structured community-based interventions (with or without medical therapy) as this can improve engagement with alcohol services and support change in harmful drinking behaviour.

## Introduction

There is a large burden of undiagnosed but clinically significant liver disease in otherwise asymptomatic but at-risk populations.^1^ This is likely to be the case in community alcohol services where high levels of physical and psychological dependence on alcohol are frequent.^2^ The 2020 Lancet Commission report into liver disease in the United Kingdom (UK) expressed serious concerns over rising liver-related mortality and the lack of contingency measures to effectively mitigate the grave situation.^3^ In the same year, in the UK, Dame Carol Black conducted an independent review of drug and alcohol prevention, treatment, and recovery and highlighted the unmet need of this community and emphasized the greater focus required on prevention.^4^ In the absence of effective measures, the age-standardised annual mortality due to alcohol-related liver disease (ARLD) in many high-income countries is predicted to rise by 75% over the next two decades.^5^

ARLD progresses silently; over 50% of patients are first diagnosed with liver disease after an emergency hospital admission at a stage when the scope of any medical and behavioural intervention is limited.^3^ Early diagnosis of ARLD provides an opportunity to intervene and reduce or stop alcohol intake. Alcohol cessation is known to be the most effective way of preventing liver disease progression.^6^ Systematic targeting of the lifestyle-related risk factors for liver disease in the community has been shown to enhance the detection rate of significant liver disease with the potential to link this to behavioural interventions.^5^^,^^7^

The addition of biomarker-based advice to personalised healthcare communications can enhance motivation to overcome addictive behaviour.^8^ In respiratory medicine, delivering tailored advice based on tests showing varying degrees of lung injury has been successfully used to promote smoking cessation.^9^ Including the results of point-of-care diabetes tests in patient feedback has been associated with improvement in compliance with diabetes treatment.^10^^,^^11^ Similar changes in alcohol consumption in response to advice based on non-invasive tests for liver disease have been previously reported.^2^^,^^12^ Methodological limitations such as observational design and lack of a control group make it difficult to distinguish whether the desired effect of change in drinking behaviour was truly due to non-invasive test-based advice. Data is also limited in amount, in part because tests such as transient elastography (TE) and enhanced liver fibrosis (ELF) are not widely available in community alcohol services.

Recovery narratives have been used by healthcare practitioners as an intervention to support patients’ recovery from physical and mental health problems.^13^ Sharing illness narratives can help patients to make informed choices on the selection of a specific treatment and can improve compliance.^14^ The act of sharing alcohol narratives has been an important component of the Alcoholics Anonymous (AA) 12-step programme.^15^ The use of recovery narratives is becoming established in mental health but relatively under-explored in drug and alcohol services specifically in the UK.^16^ Narratives provide access to the experiences of peers, and peer support groups can be beneficial in modifying high-risk drinking behaviour.^17^

UK Health Security Agency (UKHSA) and National Health Services (NHS) long-term plan advocate for maximising every contact with patients with a focus on preventative medicine.^18^ ARLD is twelve times more likely to present late compared to other aetiologies of liver disease.^19^ There is a pressing need to optimise existing interventions to reduce harmful alcohol intake and examine effective alternative options. This feasibility randomised control trial (RCT) aims to provide evidence to support the development of a definitive randomised control trial to test the effectiveness of transient elastography-based advice and alcohol recovery video stories (ARVS) in changing harmful drinking behaviour in community alcohol services.

## Methods

KLIFAD (**K**nowledge of **LI**ver **F**ibrosis **A**ffects **D**rinking) is a parallel design feasibility RCT.

### Ethics statement

The work presented in this paper received a favourable ethical review from the West of Scotland Research Ethics Service (WoSRES) on 20 January 2021, REC reference: 20/WS/0179. The RCT was prospectively registered (ISRCTN 16922410, 26/01/2021) and the trial protocol was published.^20^ The study start date was 02.10.2019 and end date 30.11.2022. The study data was closed after per-protocol follow-ups were completed for all participants.

Adults of age ≥18 years with a primary problem of alcohol misuse were included.^20^ The eligibility criteria are provided in the Supplementary material (Supplementary Table S1). Individuals presenting to any of the recruitment settings were screened by alcohol workers who offered information on the KLIFAD trial and assessed eligibility. Eligible patients were offered trial participation, informed consent was taken, and were randomised.

### Randomisation

Participants were individually allocated on a one-to-one ratio using minimisation with a probabilistic element. The minimisation variables were age (18–39, 40–59, 60–79, ≥80), gender (male, female), ethnicity (white, minority), and severity of alcohol misuse based on the SADQ score (not dependent 0–7, mild dependence 8–15, moderate dependence 16–30, severe dependence 31–60). To protect against selection bias, an online randomisation tool, REDCap cloud (version 1.6), was used and the randomisation was externally performed by a data manager from Nottingham Recovery Network not directly involved in the study process. The access was restricted to the chief investigator (SDR), study coordinator (MS), and Nottingham University Hospitals (NUH) REDCap manager for monitoring purposes. These people were not involved in using the system to randomise participants. Locally at Nottingham, the REDCap cloud is hosted and supported by NUH. Due to the nature of the interventions, it was not possible to blind the participants or key alcohol workers. The trial flow charts are provided in the supplementary material (Supplementary Figs. S1 and S2).

Eligible participants were recruited at the following three community alcohol treatment settings in the East Midlands, UK.

*Community Drug & Alcohol Day-care Centre:* is a city centre-based service. The majority of individuals self-present and a minority are referred by general practitioners (GP), or hospital-based physicians and hospital alcohol care teams. The service offers structured treatment programs to people with drug and alcohol use disorders (AUD).

*Community Drug & Alcohol Inpatient Detoxification Unit:* is a 62-bed facility for individuals who experience physical and mental health problems due to drug and alcohol use disorder. In addition to providing mental health and social support, the centre also provides a structured alcohol detoxification program.

*Primary care substance use disorder clinic*: is a GP run service. Individuals with drug and alcohol addiction are initially screened by a GP to assess their suitability for the clinic. Once in the substance use disorder clinic, the individuals are seen by drug and alcohol support workers and a GP with a special interest in the management of drugs and AUD.

As standard, the settings provide different types of interventions in line with the National Drug Treatment Monitoring System (NDTMS) and Public Health England (PHE) now UKHSA guidelines.^21^ There are three main categories of standard interventions.

a) Psychological: which includes motivational, family and social network, and cognitive and behavioural based relapse prevention interventions (substance misuse specific).
b) Recovery Support: This includes 12-step alcohol treatment programs and counselling.^22^
c) Pharmacological: which involves prescribing medication for drug and/or alcohol relapse prevention support. For example, naltrexone, acamprosate, disulfiram as part of alcohol or opioid relapse prevention therapy and Chlordiazepoxide for acute alcohol withdrawal.

Specific treatment programmes are started after an initial assessment. The programmes are individualised based on the participant's needs with most lasting for a median duration of five months and only a minority of clients staying in treatment longer than six months.

### Sample size

The researchers have previously recommended sample sizes between 24 and 50 to satisfactorily achieve feasibility outcomes.^23^ Inline with recommendations for sample size calculation for feasibility studies,^24^ and after discussion with the community alcohol services data manager and considering variation in the number of patients presenting per week, we estimated a sample size of 120. We aimed to approach 40 eligible participants per month. Assuming a 50% consent rate we anticipated randomising 20 participants per month (10 per month per arm) for a recruitment period of six months. With the planned sample size, we would be able to calculate a dropout rate of 80% within a 95% confidence interval of ± 7.1%. Assuming a non-differential follow-rate of 80%, we anticipated this target sample size should provide follow-up outcome data on a minimum of 48 participants in each of the two arms.

### Control group

Participants randomised to the control group continued with standard treatment (usual care) provided at the three treatment settings. The participants in this arm were offered transient elastography (TE) at least six months after their recruitment date after completing the follow-up measures.

### Intervention group

Participants randomised to the intervention group in addition to usual care had liver stiffness measured by TE, followed by scripted feedback based on liver stiffness measure results. The scripts for TE were created in consultation with the Patient Public Involvement (PPI) group covering three ranges of transient elastography scores, normal, intermediate fibrosis and advanced fibrosis (Supplementary Fig. S3).^25^ The participants were then provided with a catalogue of alcohol recovery video stories (ARVS) and were allowed to watch one or more ARVS of their choice on a handheld electronic device at the services.^25^ If participants were unable to watch ARVS on the same day due to any reason, they were allowed to return to services as frequently as they wished, to watch the ARVS. TE scores were defined as, normal ≤7 Kilopascal (kPa), intermediate fibrosis 8–14 kPa and advanced fibrosis ≥15 kPa.^26^

### Schedule of visits

#### Baseline

Baseline visit was the day when the participant returned to start standard treatment at any of the recruitment settings. Participants in both arms had an initial detailed assessment as part of their standard care. This included the collection of baseline demographic and clinical data (Table 1). All participants started usual care; in addition, the intervention arm attended a further appointment to have TE followed by standardised script feedback with access to ARVS immediately after receiving liver stiffness measure (LSM) results.

**Table 1:** Baseline characteristics of participants (intention to treat).

	Control group (n = 57)	Intervention group (n = 71)	Total (n = 128)
Age (mean)	44.4 ± 10.6	43.6 ± 12.9	43.9 ± 11.9
Gender			
Male	44 (77.2 [66.3–88.1])	50 (70.4 [59.8–81.0])	94 (73.4 [64.9–80.8])
Female	13 (22.8 [11.9–33.7])	21 (29.6 [19.0–40.2])	34 (26.6 [19.2–35.1])
Sexuality			
Heterosexual	52 (91.2 [83.9–98.6])	63 (90.0 [83.0–97.0])	115 (90.6 [83.3–94.5])
LGBTQ+	5 (8.8 [1.4–16.1])	7 (10.0 [3.0–17.0])	12 (9.4 [4.9–15.8])
Missing	0	1	1
Religion			
None	40 (70.2 [58.3–82.1])	47 (67.1 [56.1–78.1])	87 (68.5 [59.2–75.9])
Christian	12 (21.1 [10.5–31.6])	17 (24.3 [14.2–34.3])	29 (22.8 [15.7–30.8])
Other	5 (8.8 [1.4–16.1])	6 (8.6 [2.0–15.10])	11 (8.7 [4.3–14.9])
Missing	0	1	
Ethnic Origin			
White	46 (80.7 [70.5–90.9])	60 (84.5 [76.1–0.93])	106 (82.8 [75.1–88.9])
Minority ethnicity	11 (19.3 [9.1–29.5])	11 (15.5 [7.1–23.9])	22 (17.2 [11.1–24.9])
Disability			
Yes	15 (26.8 [15.2–38.4])	14 (20.0 [10.6–29.4])	29 (22.8 [15.7–30.8])
None	41 (73.2 [61.6–84.8])	56 (80.0 [70.6–89.4])	97 (76.4 [67.4–82.9])
Missing/Not stated	1	1	1
Mental health comorbidity			
Yes	44 (77.2 [66.3–88.1])	53 (74.7 [64.5–84.8])	97 (75.8 [67.4–82.9])
None	13 (22.8 [11.9–33.7])	18 (25.3 [15.2–35.5])	31 (24.2 [17.1–32.6])
Housing problem			
Yes	8 (14.0 [5.0–23.1])	10 (14.1 [6.0–22.2])	18 (14.1 [8.5–21.3])
No	49 (86.0 [76.9–95.0])	61 (85.9 [77.8–94.0])	110 (85.9 [78.7–91.5])
Employment status			
Employed	20 (35.1 [24.2–49.9])	29 (40.8 [29.9–53.0])	49 (39.5 [30.8–48.7])
Unemployed	12 (21.1 [11.1–33.3])	15 (21.1 [11.8–31.0])	27 (21.8 [14.9–30.1])
Long term sick or disabled	20 (35.1 [24.2–49.9])	18 (25.4 [15.5–36.0])	38 (30.6 [22.7–39.6])
Student	1 (1.8 [0.1–5.4])	5 (7.0 [1.1–13.2])	6 (4.8 [1.8–10.2])
Retired	1 (1.8 [0.1–5.4])	3 (4.2 [0.5–9.0])	4 (3.2 [0.8–8.0])
Other/Not stated	3	1	4
Poly drug use			
Yes	15 (26.3 [14.9–37.7])	20 (28.2 [17.7–38.6])	35 (27.3 [19.8–35.9])
No	42 (73.7 [62.3–85.1])	51 (71.8 [61.4–82.3])	93 (72.7 [64.1–80.2])
Drinking days (month)			
	23.3 ± 7.8	22.6 ± 7.8	22.9 ± 7.8
Daily alcohol intake			
Daily (units)	28 (18–39)	24 (17–37)	26 (17–37)
AUDIT score (median)			
	32 (26–37)	31 (28–36)	32 (27–36)
SADQ SCORE			
Non-dependent (0–7)	4 (7.0 [0.4–13.6])	4 (5.7 [0.3–11.2])	8 (6.3 [2.7–12.0])
Mild dependence (8–15)	7 (12.3 [3.8–20.8])	9 (12.9 [0.5–20.7])	16 (12.6 [7.3–19.6])
Moderate dependence (16–30)	16 (28.1 [16.4–39.7])	18 (25.7 [15.5–36.0])	34 (26.8 [19.3–35.4])
Severe Dependence (31–60)	30 (52.6 [39.7–65.6])	40 (56.7 [45.5–68.7])	69 (54.3 [45.3–63.2])
Missing/Not stated	0	1	1

Data are in mean (SD), n (% [95% CI of percentage]), median (IQR).

##### Six months

All participants were offered an in-person or telephone follow-up at six months which was undertaken by the research team. This was to engage participants who were no longer in services (discharged early or dropped out). A research support worker unaware of group allocation contacted participants and arranged the follow-up call or face-to-face appointment with the researchers (MS or HK) who remained blinded to randomisation. Before a participant was defined as lost to follow up a minimum of three separate contacts were attempted at least two days apart. Participants in the control arm were offered TE after the completion of outcomes. Semi-structured interviews were also conducted involving trial participants and feedback was taken from key alcohol workers. Follow-up data on self-reported alcohol intake, AUDIT, and completion of allocated treatment programs was collected. The schedule of the visits is summarised in the supplementary material (Supplementary Table S2).

### Outcomes

Outcomes were measures of the feasibility of conducting a national RCT. To establish the feasibility of an RCT, recruitment and retention rates were collected. The study also allowed an estimate of the potential effectiveness of the intervention to be estimated.

### Statistical analysis plan

As per CONSORT reporting guidelines and to reflect the true effect of KLIFAD intervention intention-to-treat (ITT) and per-protocol (PP) analyses were conducted.^27^^,^^28^ We reported the rates of responses and data completeness of potential primary and secondary outcomes for the future definitive RCT. In PP analysis we first restricted the analysis to participants who had TE and may or may not watch ARVS and then to participants who had TE and watched ARVS. Due to the well-known association between dual diagnosis of mental health comorbidity and AUD with poor outcomes,^29^ a dedicated analysis was performed to determine the impact of mental health comorbidity on the uptake of KLIFAD intervention and engagement with services.

Data were summarised using frequency (%), mean (SD) or median (IQR) depending on the distribution of the data. The correlation between normally distributed quantitative variables was assessed by parametric tests (Pearson's correlation coefficient, T-test, ANOVA test) and non-normally distributed by non-parametric tests (Spearman's correlation coefficient, Mann–Whitney U test). Categorical variables were analysed by the Chi-Squared test, with results reported as absolute and relative frequencies ±95% confidence interval. Summary measures are presented along with their 95% confidence intervals whenever appropriate. To calculate the change in self-reported alcohol intake and AUDIT, the change for each individual was first computed and the resulting changes were summarised. The results of the feasibility variables are presented by categories of different variables (age, gender, ethnicity, the severity of alcohol misuse)^30^ The funder of the study had no role in study design, data collection, data analysis, data interpretation, or writing of the report. A combination of STATA (version 15.64 bit) and Prism GraphPad Prism (version 9) was used in the data analysis.

### Impact of the Covid-19 pandemic

Our trial protocol described recruitment from community alcohol clinics run by Nottingham recovery networks as a third site. Due to the Covid-19 pandemic, the clinics were closed and remained closed until the completion of the study. They were replaced with General Practitioner (GP) run substance use disorder clinic as an additional site, with approval from the trial management group, trial steering committee, sponsor, and Health Research Authority (through a minor amendment). A Conserve checklist has been completed.

### Role of funding

The views expressed are those of the author(s) and not necessarily those of the funder (NIHR or the Department of Health and Social Care). The funder of this study had no role in the study design, data collection, data analysis, data interpretation, or writing of the manuscript.

## Results

**Fig. 1:**
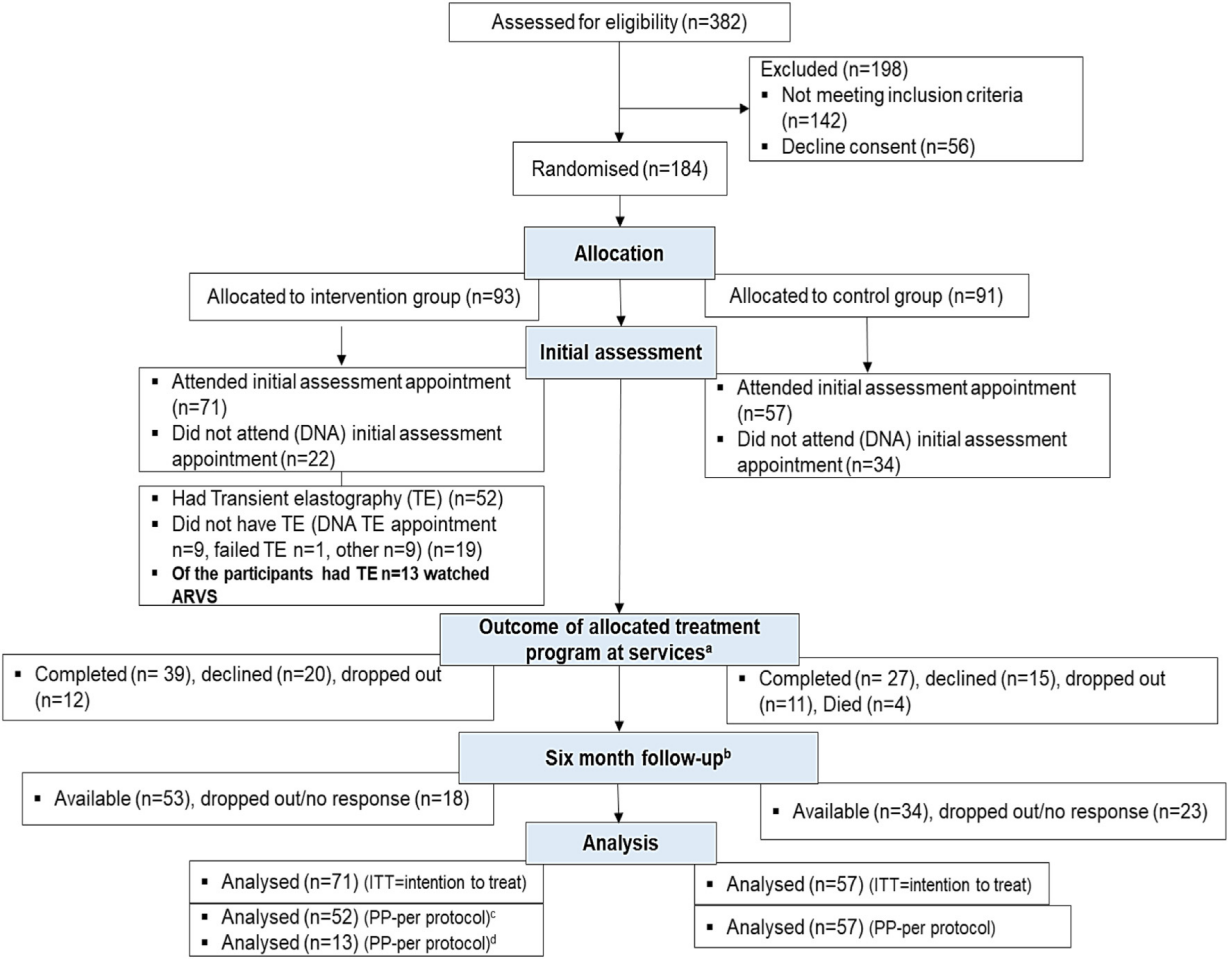
Consort flow diagram. ^a^These were routinely provided treatment programmes as part of usual care at community alcohol services. ^b^This was to engage participants who were no longer in services such as those who were discharged early or dropped out. ^c^Per-protocol analysis for participants who had transient elastography ± watched alcohol recovery video stories (ARVS). ^d^Per-protocol analysis for participants who had transient elastography and watched ARVS.

A total of 382 individuals were assessed for eligibility in three recruitment settings. Of them, 184 patients were randomised (intervention group *n* = 93, control group *n* = 91). Of randomised patients, 128 (intervention group *n* = 71, control group = 57) attended post-randomisation baseline appointments and were included. Six-month follow-up was available in 59.6% (*n* = 34) of participants in the control group and 74.6% (*n* = 53) in the intervention group. The detailed breakdown of enrolment and follow-up is provided in the consort flow diagram (Fig. 1).

### Baseline characteristics of the study population

Mean age was 43.9 years (*SD* = 11.9), and the majority identified as male (*n* = 94, 73.4%), white (*n* = 106, 82.8%), and heterosexual (*n* = 115, 89.8%). The self-reported alcohol intake at baseline was 22.9 days (SD = 7.8) per month and 26 units (IQR 17–37) per day. On the AUDIT assessment, 92.9% (*n* = 119) had possible alcohol dependence. On SADQ, 54.3% (*n* = 69) had severe alcohol dependence. Over a quarter were using substances other than alcohol (*n* = 25, 27.35%), 22.8% (*n* = 29) had a co-existing disability, 75.8% (*n* = 97) had a mental health comorbidity and 39.5% (*n* = 49) were employed. The baseline characteristics for ITT analysis are given in Table 1 and for PP analysis in the supplementary material (Supplementary Table S3). Of the 52 participants who had transient elastography, 21.2% (*n* = 11) had raised LSM (≥8 kPa) (Supplementary Fig. S4).

Participants were more likely to attend the post-randomisation baseline appointment if they were of minority ethnicity (77.8%, 95% CI 62.1–93.5) compared to white (68.2%, 95% CI 60.9–75.4), non-alcohol dependent (SADQ 0–7) (81.8%, 95% CI 59.9–99.8) compared alcohol-dependent groups (SADQ 8–15: 69.6% 95% CI 50.8–88.4, SADQ 16–30: 68.1%, 95% CI 54.8–81.4, SADQ 31–60 68.9%, 95% CI 60.0–77.9), and engaged in treatment at a Community Drug & Alcohol Inpatient Detox Unit (100%) or Primary care run substance use disorder clinic (100%) compared to Community Drug & Alcohol Day-care Centre (67.1%, 95% CI 60.9–75.4) (Supplementary Table S4). The recruitment and retention rates for individual services are given in the supplementary materials (Supplementary Table S5).

The characteristics of participants who had the KLIFAD intervention compared to those who did not are provided in the supplementary material (Supplementary Table S6). The participants with mental health comorbidity compared to those without were less likely to have TE (69.8% vs 83.3%) and complete the allocated treatment program at services (33.0% vs 58.1%) (Supplementary Table S7).

### Intervention versus control group

The baseline characteristics, mean age, median drinking days per month, daily self-reported alcohol intake, and distributions for gender, sexual orientation, religion, ethnic origin, disability, mental health comorbidity, housing problems, employment status, substance use other than alcohol, AUDIT categories, and SADQ were similar between intervention and control groups (Table 1).

### Intention to treat analysis (ITT)

#### Completion of the allocated treatment program at services

Mean duration of engagement with services for the intervention group was 159.0 days (*SD* = 106.4) and for the control group was 150.4 days (*SD* = 100.2) (mean difference 8.6 days *SD* = 18.4) (Supplementary Fig. S5). In the control group, 43.9% (*n* = 25) completed the allocated treatment program by either reducing alcohol intake or stopping drinking, 19.3% (*n* = 11) dropped out, 26.3% (*n* = 15) declined treatment, and 7.0% died (*n* = 4). Whereas in the intervention group, 54.9% (*n* = 39) completed the allocated treatment program by either reducing alcohol intake or stopping drinking, 16.9% (*n* = 12) dropped out, and 28.2% (*n* = 20) declined treatment. There were no Serious Adverse Events (Supplementary Table S8).

In the intervention group, 14.1% (*n* = 10) were still active in service at the end of the trial (stopped drinking *n* = 5, 7.0%, reduced alcohol intake *n* = 5, 7.0%, increased alcohol intake *n* = 0). In the control group, 10.5% (*n* = 6) were still active in service at the end of the trial (stopped drinking n = 4, 7.0%, reduced alcohol intake *n* = 0, increased alcohol intake *n* = 2, 3.5%) (Table 2).

**Table 2:** Completion of allocated treatment program at services.

Intention to treat	Control group (n = 57)	Intervention group (n = 71)	Difference in proportion [95% CI], (control–intervention)
Incomplete died	4 (7.0 [2.7, 16.7])^a^	0	7.0 [0.4, 13.6]
Incomplete dropped out	11 (19.3 [11.1, 31.3])	12 (16.9 [9.9, 27.3])	2.4 [–11.1, 15.8]
Incomplete declined	15 (26.3 [16.6, 39.0])	20 (28.2 [19.0, 39.5])	−1.9 [−17.4, 13.6]
Completed alcohol free^b^	9 (15.8 [8.5, 27.4])	14 (19.7 [12.1, 30.4])	−3.9 [−17.2, 9.3]
Completed occasional alcohol user^c^	12 (21.1 [12.5, 33.3])	15 (21.1 [13.2, 32.0])	0 [−14.3, 14.1]
Active in services alcohol free	4 (7.0 [2.7, 16.7])	5 (7.0 [3.0, 15.4])	0 [−8.9, 8.9]
Active in service occasional alcohol user	0	5 (7.0 [3.0, 15.4])	−7.0 [−13, −1.1]
Active in service increased alcohol intake	2 (3.5 [1.0, 11.9])	0	
**Per-protocol**	**Received TE (n = 52)**^d^	**Received TE + watched ARVS (n = 13)**^e^	

Incomplete died	0	0	0
Incomplete dropped out	6 (11.5 [5.4, 23.0])	2 (15.4 [4.3, 42.2])	−3.9 [−25.3, 17.6]
Incomplete declined	11 (21.2 [12.2, 34.0])	3 (23.1 [8.2, 50.3])	−1.9 [−27.4, 23.5]
Completed alcohol free	13 (25.0 [15.2, 38.2])	2 (15.4 [4.3, 42.2])	9.6 [−13.3, 32.5]
Completed occasional alcohol user	13 (25.0 [15.2, 38.2])	4 (30.8 [12.7, 57.6])	−5.8 [−33.5, 21.9]
Active in services alcohol free	5 (9.6 [4.2, 20.6])	0	9.6 [1.6, 17.6]
Active in service occasional alcohol user	4 (7.7 [3.0, 18.2])	2 (15.4 [4.3, 42.2])	−7.7 [−28.6, 13.2]
Active in service increased alcohol intake	0	0	0

Data are number (% [95% CI]); TE, Transient elastography; ARVS, Alcohol recovery video stories.

a None of deaths were related to study procedures.

b Alcohol free: participants stopped drinking alcohol.

c Occasional alcohol user: participant report reduction in alcohol consumption.

d Participants had transient elastography and received feedback based on liver stiffness measure results (LSM) may or may not watched alcohol recovery video stories (ARVS).

e Participants had transient elastography and received feedback based on liver stiffness measure (LSM) results and watched ARVS.

#### Change in self-reported alcohol at six months

A summary of the change in self-reported alcohol intake measures is given in Table 3.

**Table 3:** Change in self-reported measures of alcohol at six month follow up^a^ (table of efficacy).

Intention to treat	Control group (n = 34)	Intervention group (n = 53)
Change in drinking days (mean)	−12.1 ± 12.9	−11.9 ± 10.8
Change in daily units (median)	−17.5 (−32.0, −8.1)	−20.0 (−29.2, −11.0)
Change in AUDIT (median)	−12.0 (−20.5, −0.8)	−20.0 (−27.0, −10.5)
**Per-protocol**	**Received TE (n = 42)**^b^	**Received TE and watched ARVS (n = 9)**^c^

Change in drinking days (mean)	−11.9 ± 10.7	−6.7 ± 10.2
Change in daily units (median)	−22.5 (−31.5, −12.0)	−17.0 (−27.0, −11.1)
Change in AUDIT (median)	−19.5 (−28.0, −12.0)	−21.0 (−24.0, −15.0)

Data are given in mean (SD), median (inter quartile range -IQR); TE, Transient elastography; ARVS, Alcohol recovery video stories.

a This was to particularly capture participants who were no longer in services such as those who were discharge early or dropped out.

b Participants had transient elastography and received feedback based on liver stiffness measure results (LSM) may or may not watched ARVS.

c Participants had transient elastography and received feedback based on liver stiffness measure (LSM) results and watched ARVS.

##### Reduction in drinking days per month (4 weeks) and daily alcohol consumption (units)

Mean change in drinking days by the control group was −12.1 (*SD* = 12.9) days per month compared to −11.9 (*SD* = 10.8) days per month in the intervention group (Table 3 & Supplementary Fig. S6).

Median reduction in daily alcohol consumption by the control group was −17.5 (IQR −32.0, −8.1) units per day compared to −20.0 (IQR −29.2, −11.0) units per day in the intervention group (Table 3).

##### Reduction in AUDIT

The control group had a median change of −12.0 (IQR −20.5, −0.8) in AUDIT score compared to −20.0 (IQR −27.0, −10.5) in the intervention group (Supplementary Fig. S6 and Table S3).

Based on the audit score at baseline alcohol dependence fell from 91.2% (*n* = 52) to 32.4% (*n* = 11) in the control vs 94.4% (*n* = 67)–26.4% (*n* = 14) in the intervention group. The fall to AUDIT-C scores no longer representing a diagnosis of AUD was seen in 20.6% (*n* = 7) in the control group vs 41.5% (*n* = 22) in the intervention group. Four participants in the control group and two in the intervention group had an increase in AUDIT score. Out of these only in a single participant from the intervention group, the increment in the AUDIT score resulted in an increase in the AUDIT by one category (Table 4).

**Table 4:** Change in AUDIT category at six month follow up^a^ (ITT).

AUDIT Category at baseline	Control group (n = 57)	Intervention group (n = 71)	Difference in proportion [95% CI], (control–intervention)
Low Risk	0	0	0
Increasing risk	2 (3.5 [0.1, 11.9])	1 (1.4 [0.2, 7.6])	2.1 [−3.4, 7.6]
High Risk	3 (5.3 [1.8, 14.4])	3 (4.2 [1.4, 11.7])	1.1 [−6.4, 8.5]
Possible dependence	52 (91.2 [81.1, 96.2])	67 (94.4 [86.4, 97.8])	−3.2 [−12.2, 6.0]
**AUDIT Category at follow-up (6-month)**	**Control group (n = 34)**	**Intervention group (n = 53)**	

Low Risk	7 (20.6 [10.3, 36.8])	22 (41.5 [29.3, 54.9])	−20.9 [−40.0, −1.9]
Increasing risk	13 (38.2 [23.9, 55.0])	11 (20.8 [12.0, 33.5])	17.4 [−2.2, 37.1]
High Risk	3 (8.8 [3.0, 23.0])	6 (11.3 [5.3, 22.6])	−2.5 [−15.3, 10.3]
Possible dependence	11 (32.4 [19.1, 49.2])	14 (26.4 [16.4, 39.6])	6 [−13.8, 25.6]
Missing data	23	18	
**Change in AUDIT category from baseline**	**Control group (n = 34)**	**Intervention group (n = 53)**	

No change	13 (38.2 [23.9, 55.0])	14 (26.4 [16.4, 39.6])	11.8 [−8.4, 32.0]
AUDIT down in category	21 (61.8 [45.0, 76.1])	38 (71.7 [58.4, 82.0])	−9.9 [−30.3, 10.4]
AUDIT up in category	0	1 (1.9 [0.3, 9.9])	−1.9 [−5.5, 1.8]
Missing data	23	18	
**Scale of change in AUDIT category from baseline**	**Control group (n = 34)**	**Intervention group (n = 53)**	

No change	13 (38.2 [23.9, 55.0])	14 (26.4 [16.4, 39.6])	11.8 [−8.4, 32.0]
AUDIT increased by one category	0	1 (1.9 [0.3, 9.9])	−1.9 [−5.5, 1.8]
AUDIT reduced by one category	5 (14.7 [6.4, 30.1])	5 (9.4 [4.1, 20.3])	5.3 [−9.0, 19.5]
AUDIT reduced by two categories	10 (29.4 [16.8, 46.2])	12 (22.6 [13.5, 35.5])	3.8 [−12.2, 25.8]
AUDIT reduced by three categories	6 (17.6 [8.3, 33.5])	21 (39.6 [27.6, 53.1])	−22 [−40.3,−3.6]
Missing data	23	18	

Data are number (% [95% CI]).

a This was to particularly capture participants who were no longer in services such as those who were discharge early or dropped out.

### Per-protocol analysis

Of the participants allocated to the intervention group 52 had TE plus feedback based on LSM and 13 had TE plus feedback based on LSM and watched ARVS. Length of appointment and not being able to access ARVS at home were the most common reason for not accessing the videos.

#### Participants who had transient elastography

At baseline, the participants in this group reported drinking for 22.0 (*SD* = 8.2) days per month consuming 26 units (IQR 18–38) per day. The median AUDIT score at baseline was 32 (IQR 28–36) (Supplementary Table S3).

##### Completion of the allocated treatment program at services

Mean duration of engagement with services for the participants who had transient elastography was 180.1 days (*SD* = 107.1) (Supplementary Fig. S5) and the follow-up rate at six months was 80.8% (*n* = 42) (Supplementary Table S3).

On restricting the analysis to the participants who had transient elastography (*n* = 52), 67.3% (*n* = 35) completed the allocated treatment program by either reducing alcohol intake or stopping drinking, 11.5% (*n* = 6) dropped out, and 21.2% (*n* = 11) declined treatment, 17.3% (n = 9) were still active in service at the end of the trial (stopped drinking *n* = 5, 9.6%, reduced alcohol intake *n* = 4, 7.7%) (Table 2).

##### Change in self-reported alcohol measures at six months

At six months the participants who had TE reduced drinking days by a mean of −11.9 (*SD* = 10.7) days per month, daily alcohol consumption by a median of −22.5 (IQR −31.5, −12.0), and AUDIT score by a median of −19.5 (IQR −28.0, −12.0) (Table 3). Based on the audit score at baseline alcohol dependence fell from 92.3% (*n* = 48) to 21.4% (*n* = 9) (Supplementary Table S9). One of the participants who received TE reported an increase in the AUDIT category.

#### Participants who had TE and watched ARVS vs those who had TE but did not watch ARVS

Mean age of participants who had TE and watched ARVS was 44.0 years (*SD* = 16), these participants reported drinking for 21.8 (*SD* = 9.6) days per month consuming 20 units (IQR 15–28) per day. The median AUDIT score at baseline was 29 (IQR 23–36). None of the participants from minority ethnicity watched ARVS (Supplementary Table S3). The characteristics of participants who had TE and watched ARVS compared to those who had TE but did not watch ARVS are given in supplementary material (Supplementary Table S10). Participants with coexisting disability and/or mental health diagnoses were more likely to watch ARVS after having TE.

##### Completion of the allocated treatment program at services

Mean duration of engagement with services for these participants was 174.1 days (*SD* = 97.89) (Supplementary Fig. S5). Among the participants who watched ARVS 61.5% (*n* = 8) completed the allocated treatment program by either reducing alcohol intake or stopping drinking, 15.4% (*n* = 2) dropped out, and 23.1% (*n* = 3) declined further treatment. In the group who did not watch ARVS 71.8% (*n* = 28) completed the allocated treatment program by either reducing alcohol intake or stopping drinking, 7.7% (*n* = 3) dropped out, and 20.5% (*n* = 8) declined further treatment (Supplementary Table S11).

##### Change in self-reported alcohol measures at six months

At six months among the participants who watched ARVS changed drinking days by a mean of −6.7 (*SD* = 10.2) days per month, daily alcohol consumption by a median of −17.0 (IQR −27, −11.1), and AUDIT score by a median of −21.0 (IQR 24.0, −15.0). In comparison among the participants who did not watch ARVS changed drinking days by a mean of −13.4 (*SD* = 10.5) days per month, daily alcohol consumption by a median of −25.0 (IQR −37.0, −12.0), and AUDIT score by a median of −19.0 (IQR −28.5, −11.5) (Supplementary Table S12). At end of six months based on AUDIT score 33.3% (*n* = 3) participants who watched ARVS compared to 42.4% (*n* = 14) of those who watched ARRVS had no AUD (Supplementary Table S13).

#### Effect of clustering

As this is a feasibility study with no consideration of clustering at the design level of the study, our analysis is limited to descriptive statistics. An analysis to identify the effect of clustering by the three services was undertaken and some baseline results are provided in supplementary material (Supplementary Tables S14 and S15).

## Discussion

The study demonstrated that the integration of non-invasive testing of liver stiffness by transient elastography into community alcohol services is feasible. Of the eligible individuals, 77% gave informed consent, 51.6% completed the allocated treatment program at services, and six-month trial specific follow-up was available in 68%. Initial assessment data were available for 76% (71 out of 93) of participants allocated to the intervention group and for 63% (57 out of 91) for those allocated to the control group. The study was conducted during the Covid-19 pandemic, as contingency measures the standard operating procedures for the community alcohol services that we worked with had been amended in line with national guidelines. The initial screening visit which was face to face pre-pandemic was switched to virtual delivery. The initial high dropout rate possibly reflects the change in interface of services. However, the subsequent recruitment and retention rates were higher than those previously demonstrated in primary care-based studies screening for chronic liver disease^31^ and were similar to randomised control trials investigating behavioural intervention in smoking cessation.^32^ Previously reported evidence exploring the acceptability of transient elastography to screen for CLD in a UK primary care setting demonstrates TE was an acceptable intervention for patients presenting to community alcohol services.^33^

Although our paper aimed to evaluate the feasibility of a trial, our work has also detected a significant, previously unknown, burden of liver disease. In the asymptomatic but high-risk group, we showed that one in five had a raised liver stiffness measure, with one in seven in the cirrhotic range. The community burden of undiagnosed liver disease, especially in a high-risk population is an ongoing concern among the liver community.^34^ Population studies using non-invasive tests for liver disease report around 5% of the general population and 18–27% of the at-risk population have undetected significant liver fibrosis.^35^ Integration of non-invasive testing for liver disease into community alcohol services facilitates early detection of liver disease in an otherwise high-risk asymptomatic population. National Institute for Health and Care Excellence (NICE) guidelines state adults with high levels of alcohol dependency should be assessed and offered intensive structured community-based interventions (with or without medical therapy) as these provide the best chance of achieving and maintaining abstinence from alcohol.^36^

While not powered to show a statistical difference in key indicators of behaviour change related to alcohol intake, there were very promising trends shown in our study. At six months, the intervention compared to the control group was more likely to reduce or stop drinking and reduce the AUDIT category. In the intervention group, 41% reduced the AUDIT category to a level that they no longer had alcohol use disorder compared to 21% in the control group. The finding of normal or elevated liver stiffness did not lead to unintended negative effects such as an exacerbation in alcohol consumption. Our study suggests that biomarker feedback may improve outcomes and there is an increasing body of data to support this.^37^ A recent systematic review suggested providing feedback to patients based on markers of liver injury is an effective way to reduce harmful alcohol intake.^38^ A feasibility study invited 1128 individuals with hazardous or harmful drinking from nine primary care practices in the UK to have a liver fibrosis test using the Southampton Traffic Light (STL) test^15^ of them 38% (*n* = 393) attended to have STL. Follow-up AUDIT score at 8–12 months was available in 77% (303), 50% (*n* = 76/153) with evidence of liver damage reduced drinking compared to 35% (*n* = 52/150) without liver damage.^15^ A prospective cohort control study invited 156 individuals who self-identify as high-risk drinkers and presented to community alcohol services to have liver fibrosis tested using TE. Of invited individuals, 56% (*n* = 87) attended TE appointments, and 38% had raised LSM. The participants with raised LSM reduced weekly alcohol intake by 78.5 units compared to 25.0 units with normal LSM.^2^

The intervention compared to the control group had a longer duration of engagement with services (mean difference 8.6 days SD = 18.4), was more likely to attend further assessment appointments, stay in services and complete the allocated treatment program. This is an important trend, which can be examined through future work. A prospective observational study from Scotland recruited 76 participants who self-identified themselves as harmful drinkers and presented to community alcohol services, 26% (*n* = 20) had LSM and were referred for further management. The study demonstrated that the provision of TE was associated with subsequent high uptake both in nurse lead and specialist liver clinics.^39^ The evidence supports that engagement with services and length of time spent in drug and alcohol services are associated with favourable post-treatment outcomes.^40^

This is the first RCT to demonstrate the feasibility of the addition of feedback based on liver stiffness measures and alcohol recovery video stories besides usual care in community alcohol services. Alcohol treatment services are an ideal setting for early diagnosis of ARLD and interventions to prevent further physical harm. There is a large burden of undiagnosed liver disease in the community. Integrating non-invasive liver stiffness testing into these services creates an opportunity to change the natural history of disease progression in high-risk individuals.^3^ Most individuals who attend community alcohol services have self-presented and often have high levels of dependency; this suggests that even in the most difficult groups to treat from an alcohol perspective this intervention will help.

This study does have limitations. The patient sample may not be representative of all harmful drinkers. Due to the nature of the intervention, it was not possible to blind the participant to the intervention. The other limitations include a study conducted in one geographical area, the East Midlands (UK), with a predominantly white ethnic distribution. The study was to demonstrate the feasibility of our study design and the acceptability of the intervention, a future definitive large-scale RCT would aim to be more inclusive to overcome these limitations. Only 25% (*n* = 13) of participants who had TE watched the ARVS. A full analysis of process evaluation interviews with participants and key alcohol workers will be reported elsewhere, but preliminary analysis suggests the length of the appointment and restricted access to ARVS only at recruitment services were the most common reasons for not watching ARVS. Studies from mental health settings have demonstrated that mental health recovery narratives can be integrated into web-based interventions.^41^ There is evidence that making such ARVS available online to view when the client has both times and is in the best place psychologically to benefit may be helpful and overcomes some of these access issues and we aim to explore out-of-clinic provision of access to ARVS in our future studies.^16^ Among those with an initial assessment, a reasonably comparable proportion (55% in the intervention group and 47% in the control group) of them had complete outcome data on the allocated treatment program at services within three months of the initial assessment. However, the extent of data completeness between the two trial arms widens at the 6-month follow-up (75% and 60% available for the intervention and control, respectively). We assume the provision of trial intervention improved engagement. Learning from this feasibility study we will consider adopting evidence-based strategies such as monetary incentives, involving families, and creating feedback loop to further increase retention in definitive RCT.^42^ The missing values might have an impact on the comparison between the two trial arms with respect to some measures (e.g., the response rate). However, we feel that some of the underlying reasons for the discrepancy were related to Covid-19, and other reasons will be discussed and solved when designing the future definitive trial.

Almost two-thirds of our trial participants had concomitant mental health comorbidities. Previous epidemiological studies have demonstrated a high prevalence of mental health illness in patients with AUD and subsequent poorer outcomes.^29^ The lifetime prevalence of AUD in major depressive disorders ranges from 27% to 40%, anxiety disorders range from 20 to 40%, and post-traumatic stress disorders range from 34% to 55%.^29^ This demonstrates the importance of integrated treatment strategies addressing both AUD and mental health which have been reported to improve outcomes.^43^ There needs to be adequate service provision to deal with both alcohol problems and any underlying mental health disorders as stated by The Royal College of Psychiatry UK.^44^

In our study self-presentation was the most common source of referral, the evidence shows this group of patients are more likely to engage with health promotion programmes.^45^ Self-motivation has been widely shown to be an independent factor in behavioural change, and self-motivated people are more likely to sustain long-term recovery from substance misuse.^46^ In a study investigating the performance of non-invasive tests for liver fibrosis in people who are homeless, the researchers demonstrated self-motivations was associated with an increase in the uptake of health interventions.^47^ Self-motivation can be modified. The evidence supports that this patient subset should be the focus of action-oriented behavioural intervention programmes including in managing AUD.^45^

The study has demonstrated the successful feasibility of conducting an RCT to test the effectiveness of advice based on non-invasive tests for liver disease and ARVS compared to usual care. Based on the finding from this trial the study team have been successful in obtaining further NIHR funding (NIHR155469). Through this funding we aim to build sustainable partnerships with alcohol treatment services for delivering high-quality randomised control trials testing the efficacy of liver disease biomarker-based behavioural interventions in reducing alcohol-related harm.

The finding support integration of TE into community alcohol services both for early diagnosis of liver disease and to supplement the desired behaviour change. Previous studies have demonstrated in the community by systematic targeting of the risk factors for liver disease (hazardous alcohol intake and type-2-diabetes) significantly enhance the detection rate of liver disease.^7^ Early detection of liver disease followed by targeted interventions is a logical and effective way to reduce the risk of late presentation and to minimise alcohol-related harm. Screening patients with novel biomarkers to demonstrate significant physical damage can have an additional benefit to just detecting disease and can supplement subsequent decision making towards a healthier lifestyle.^12^ In future, we will explore people's experiences of receiving these results and going through the process, and how they felt this influenced their behaviour.

In conclusion, Integration of transient elastography in addition to usual care in community alcohol services is feasible. It can supplement the reduction in self-reported alcohol consumption, improve compliance with allocated treatment programs at services, and increase trial specific follow-up rates. One in five patients presenting to these services has a raised liver stiffness measure (LSM) at opportunistic screening. Normal or raised liver stiffness results do not provide false reassurance. Dual diagnosis of alcohol use disorder and mental health was observed in over two third of trial participants and was related to higher dropout rates. There were no reported serious adverse events related to the trial intervention. A definitive trial is indicated to evaluate the benefits of TE; a range of primary outcomes are possible, including treatment engagement, alcohol consumption, and biological measures of liver disease.

## Contributors

Mohsan Subhani.

Conceptualization, Resources, Data Curation, Software, Formal analysis, Funding acquisition, Validation, Investigations, Visualization, Methodology, Writing-original draft, Project administration, Writing-review & editing.

Holly Knight.

Validation, Visualization, Methodology, Writing-review & editing.

Katy A Jones.

Validation, Visualization, Methodology, Writing-review & editing.

Kirsty Sprange.

Conceptualization, Funding acquisition, Validation, Visualization, Methodology, Writing-review & editing.

Stefan Rennick-Egglestone.

Conceptualization, Funding acquisition, Validation, Visualization, Methodology, Writing-review & editing.

Joanne R Morling.

Conceptualization, Funding acquisition, Supervision, Writing-review & editing.

Doyo G Enki.

Data Curation, Software, Formal analysis, Validation, Visualization, Methodology, Writing-review & editing.

Andrew Wragg.

Patient and public involvement (PPI) coordinator, Conceptualization, Methodology, Writing-review & editing.

Clare Hutton.

PPI co-applicant, Conceptualization, Methodology, Writing-review & editing.

Stephen D Ryder.

Chief investigator, Conceptualization, Resources, Funding acquisition, Validation, Investigations, Visualization, Methodology, Project administration, Writing-review & editing.

Final approval of manuscript.

All authors.

Accountable for all aspects of the work.

All authors, MS, DGE, HK, KAJ, KS, SRE, JRM, AW, CH, and SDR accessed and verified all the data. All authors had access to the data in the study and had final responsibility for the decision to submit the manuscript. All authors approved the manuscript submission, confirm the accuracy and completeness of data and compliance with study protocol.

## Data sharing statement

Data supporting the findings are available upon reasonable request to the corresponding author.

## Declaration of interests

This project is funded by the 10.13039/501100000272National Institute for Health and Care Research (NIHR) under its Research for Patient Benefit (RfPB) Programme (Grant Reference Number NIHR201146). The views expressed are those of the author(s) and not necessarily those of the NIHR or the 10.13039/501100000276Department of Health and Social Care.
