# Supplementary material for: Does knowledge of liver fibrosis affect high-risk drinking behaviour (KLIFAD): an open-label pragmatic feasibility randomised controlled trial
Source: eClinicalMedicine. 2023 Jun 30;61:102069. doi: 10.1016/j.eclinm.2023.102069 (PMC10336239; doi:10.1016/j.eclinm.2023.102069)
Supplement: Supplementary Figs S1–S6 and Tables S1–S15 [file mmc1.docx]

**Supplementary material appendix**

Contents

[Sup Figure 1. KLIFAD trial work packages 2](#_Toc135904104)

[Sup Figure 2. KLIFAD feasibility RCT (WP 3) flow diagram 3](#_Toc135904105)

[Sup Figure 3. Example of a scripted feedback for advanced liver fibrosis 3](#_Toc135904106)

[Sup Figure 4. Results of transient elastography (kPa- Kilopascal, ≥15 kPa Advanced liver fibrosis, 8-14 kPa intermediate liver fibrosis, ≤7 kPa Normal/no significant liver fibrosis) 8](#_Toc135904107)

[Sup Figure 5. The mean length of engagement of participants with community alcohol services. 9](#_Toc135904108)

[Sup Figure 6. Change in measures of self-reported alcohol intake at six months 10](#_Toc135904109)

[Sup Link 1. Alcohol recovery video story of the participant who had transient elastography 11](#_Toc135904110)

[Sup Table 1. Eligibility criteria 12](#_Toc135904111)

[Sup Table 2. Schedule of visits 13](#_Toc135904112)

[Sup Table 3. Baseline characteristics of participants (per-protocol) 14](#_Toc135904113)

[Sup Table 4. Characteristics of participants who attended post randomisation baseline appointment compared to one who dropped out 15](#_Toc135904114)

[Sup Table 5. Recruitment, completion of allocated treatment and retention rates for individual services 16](#_Toc135904115)

[Sup Table 6. Comparison of the participants who had KLIFAD interventions versus those who did not 17](#_Toc135904116)

[Sup Table 7. Mental health comorbidity and uptake of KLIFAD intervention and engagement with services 18](#_Toc135904117)

[Sup Table 8. Safety reporting table 19](#_Toc135904118)

[Sup Table 9. Per-protocol analysis for change in AUDIT category at six month follow up^a^ 20](#_Toc135904119)

[Sup Table 10. Comparison of participants who watched ARVS to those who did not 22](#_Toc135904120)

[Sup Table 11. Completion of allocated treatment program at services for patient who watched ARVS vs those who did not (per-protocol analysis) 23](#_Toc135904121)

[Sup Table 12. Change in self-reported measures of alcohol at six month follow up for participants who watched ARVS vs those who did not 25](#_Toc135904122)

[Sup Table 13. Change in AUDIT for patient who watched ARVS vs those who did not (per-protocol analysis) 26](#_Toc135904123)

[Sup Table 14. Median (IQR) for a few outcomes by settings (treatment site) 27](#_Toc135904124)

[Sup Table 15. Estimated ICC by trial arm based on baseline values of few outcome variables 28](#_Toc135904125)

# Sup Figure 1. KLIFAD trial work packages


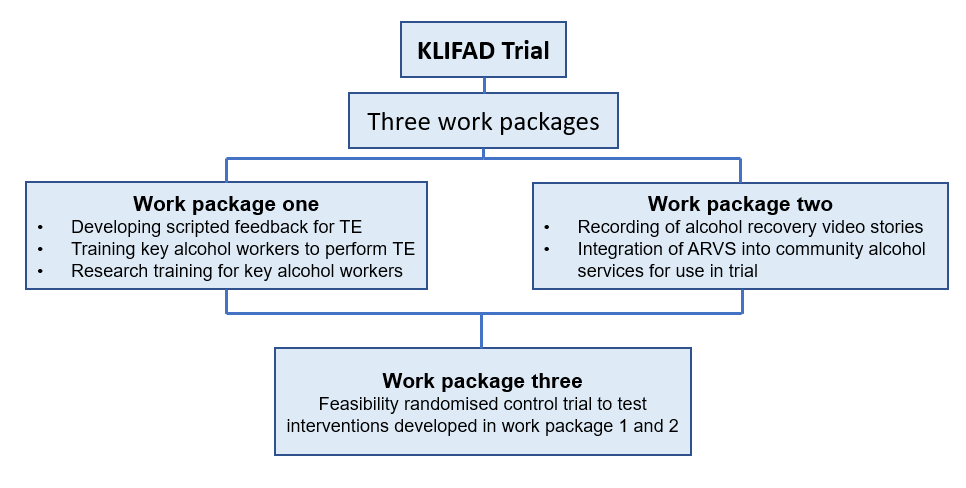


TE- Transient elastography, ARVS-alcohol recovery video stories

# Sup Figure 2. KLIFAD feasibility RCT (WP 3) flow diagram


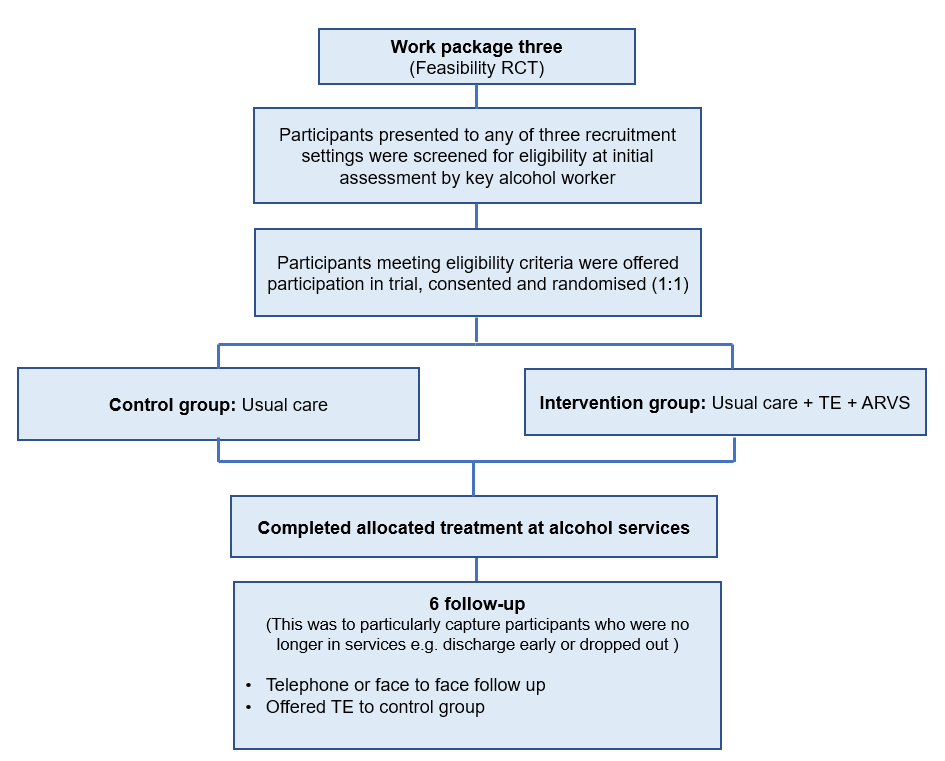


# Sup Figure 3. Example of a scripted feedback for advanced liver fibrosis

TE- Transient elastography, ARVS-alcohol recovery video stories


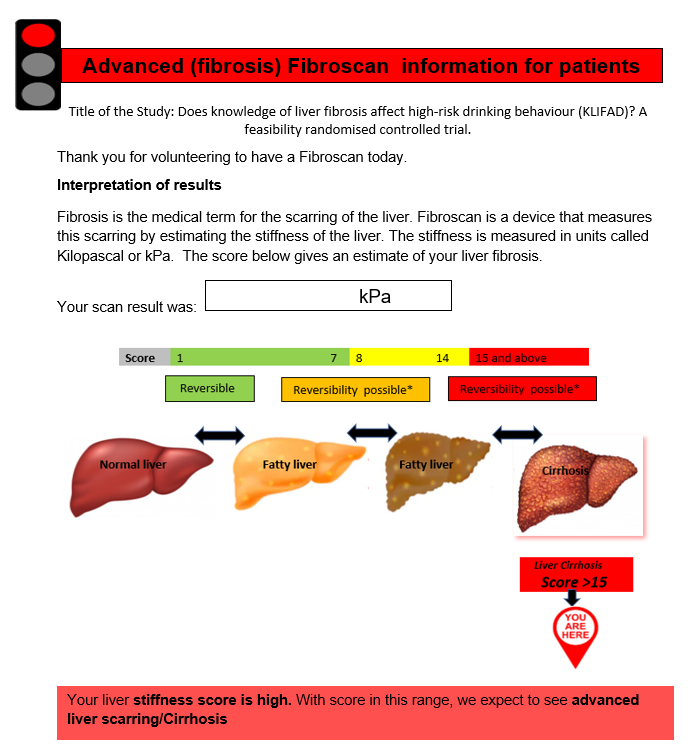


**What risk is there to my health?**

Alcohol is one of the main causes of cirrhosis. If you continue to drink at the current level and do not abstain from alcohol, you have more than **30% (1 person in 3)** chance of dying within **5 years.** Once you develop complications, the **risk of death within 5 years is as high as 65% (2 person in 3)**.

It is very important that you should stop drinking alcohol completely. **If you can stop drinking alcohol, your risk of future liver problems is significantly reduced.** If you continue to drink heavily then you are at risk of serious complications, such as liver failure as shown in next image. Liver failure has a huge impact on people, affecting their ability to live independently and increasing their risk of early death.


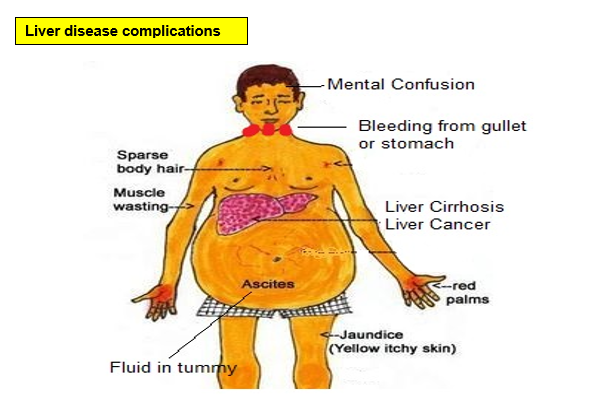


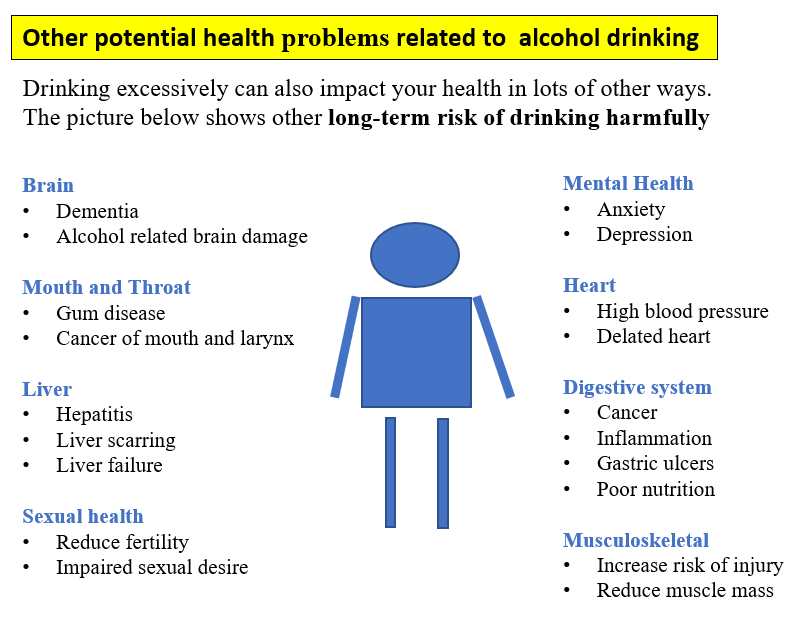


**Advice**

With liver damage is at this stage there is still a possibility of reversibility. Our advice is to stop drinking alcohol permanently as this will help your liver to function well and prevent further damage and future complications. For some people, **it may be dangerous to stop drinking suddenly**, so we advise gradually reducing the amount you drink and discussing this with your key alcohol worker or GP. We will also recommend that your GP refers you to the Nottingham University Hospital Liver team for further advice.


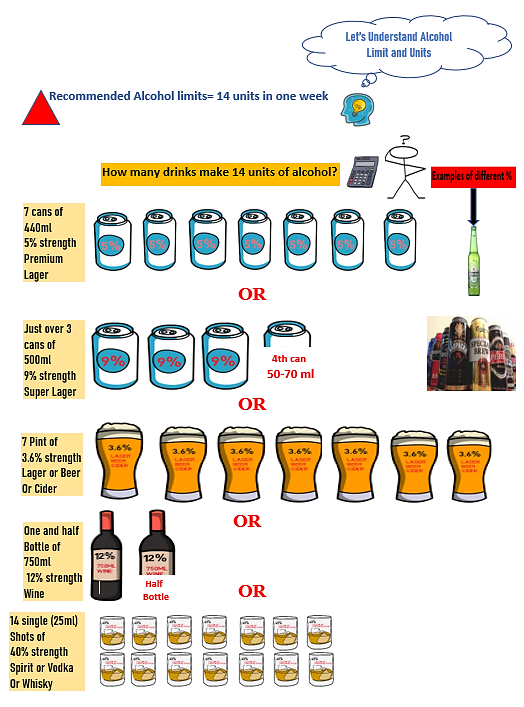


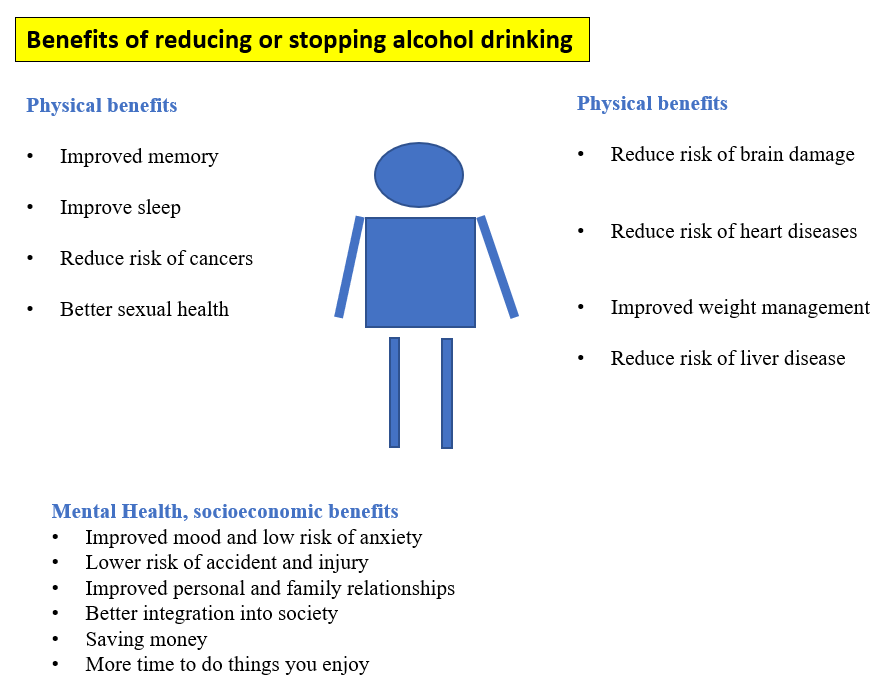

# Sup Figure 4. Results of transient elastography (kPa- Kilopascal, ≥15 kPa Advanced liver fibrosis, 8-14 kPa intermediate liver fibrosis, ≤7 kPa Normal/no significant liver fibrosis)


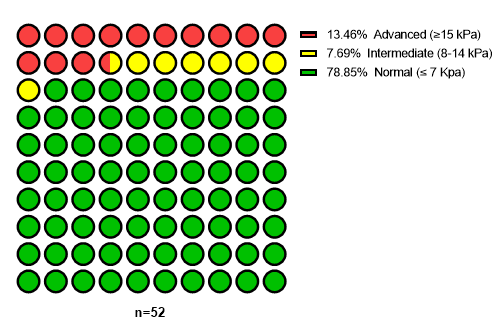


# Sup Figure 5. The mean length of engagement of participants with community alcohol services.

TE: Participants who had transient elastography and received feedback based on liver stiffness measure results (LSM) may or may not watch alcohol recovery video stories (ARVS).

ARVS_Yes: Participants who had transient elastography and received feedback based on liver stiffness measure results (LSM) and watched ARVS.

ARVS_No: Participants who had transient elastography and received feedback based on liver stiffness measure results (LSM) but did not watch ARVS.

# Sup Figure 6. Change in measures of self-reported alcohol intake at six months

**(a)** drinking days per month **(b)** daily alcohol consumption in units **(c)** Alcohol use disorder identification test (AUDIT)


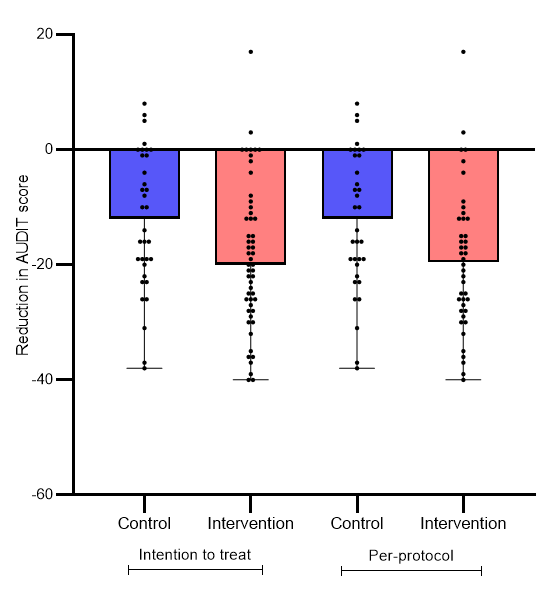

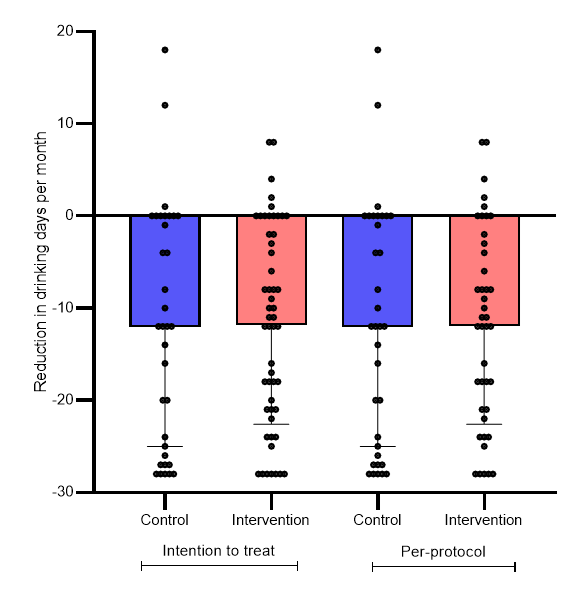

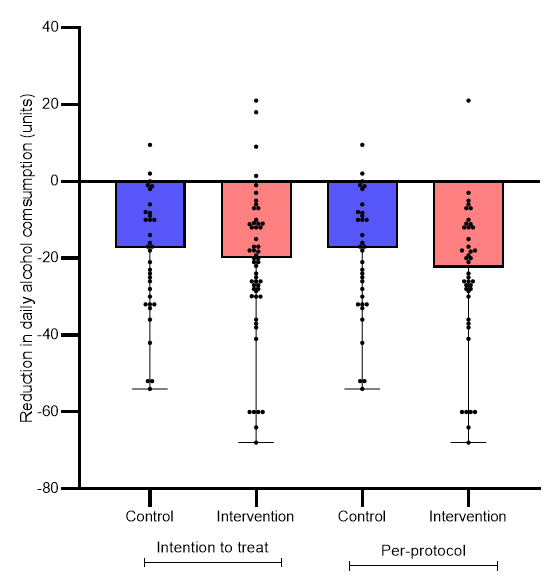


# Sup Link 1. Alcohol recovery video story of the participant who had transient elastography

URL: <https://youtu.be/mqm8kZ3xlng>

Caption: The participant has given informed consent to share the video. In this video, she shares her experience of recovery from alcohol use disorder, the adversities she faced, and the importance of knowledge on how alcohol can affect the liver.

# Sup Table 1. Eligibility criteria

| Inclusion criteria | Exclusion criteria |
| --- | --- |
| A person of age ≥18 years | Other primary substance misuses even where alcohol is a factor |
| The primary problem of alcohol misuse | Lacks the capacity to confirm consent |
|  | Referrals from driving offences and student referrals^a^ |
|  | Out-of-area clients at Edwin house^b^ |
|  | Participants unable to comply with study procedures |

^a^As these individuals are essentially not self-presenting, may have different motivations and have lower overall levels of alcohol use and so are at substantially lower risk of having liver disease
^b^In whom we cannot obtain follow-up data due to lack of follow-up availability

# Sup Table 2. Schedule of visits

| **Study Activity** | **Baseline visit** |  | **Six^a^ months** |
| --- | --- | --- | --- |
| **Control group** | | | |
| Date & Time | Yes |  | Yes |
| Baseline consent | Yes |  | - |
| Fibroscan + Feedback | - |  | Yes |
| Watching video stories | - |  | Yes |
| Demographics | Yes |  | - |
| AUDIT score | Yes |  | Yes |
| SADQ score | Yes |  | - |
| Self-reported alcohol intake^b^ | Yes |  | Yes |
| Breath alcohol test | Yes |  | - |
| Substance misuse other than alcohol | Yes |  | Yes |
| Data on feasibility outcomes | Yes |  | Yes |
| **Intervention group** | | | |
| Date & Time | Yes |  | Yes |
| Baseline consent | Yes |  | - |
| Fibroscan + Feedback | Yes |  | - |
| Watching video stories | Yes |  | - |
| Demographics | Yes |  | - |
| AUDIT score | Yes |  | Yes |
| SADQ score | Yes |  | - |
| Self-reported alcohol intake | Yes |  | Yes |
| Breath alcohol test | Yes |  | - |
| Substance misuse other than alcohol | Yes |  | Yes |
| Data on feasibility outcomes | Yes |  | Yes |

(Alcohol Use Disorder Identification Test- AUDIT, Severity of alcohol dependence questionnaire-SADQ)
^a^Six -months visit: will be a telephone consultation and/or if possible/required in person. The participant in the control group will be offered a Fibroscan at 6 months if they attend it will be an in-person appointment
^b^Self-reported alcohol intake in grams and units per week

# Sup Table 3. Baseline characteristics of participants (per-protocol)

|  | **Control group (n=57)** | **Had transient elastography (n=52)** | **Watched ARVS (n=13)** |  |
| --- | --- | --- | --- | --- |
|  |  |  |  |  |
| Age (mean) | 44.4 +/- 10.6 | 43 +/- 12 | 44+/- 16 |  |
| Gender |  |  |  |  |
| Male | 44 (77.2 [66.3-88.1]) | 36 (69.2 [56.7-81.8]) | 9 (69.2 [44.1-94.3]) |  |
| Female | 13 (22.8 [11.9-33.7]) | 16 (30.8 [18.2-43.3]) | 4 (30.8 [5.7-55.9]) |  |
| Sexuality |  |  |  |  |
| Heterosexual | 52 (91.2 [83.9-98.6]) | 46 (90.2 [82.0-98.4]) | 11 (84.6 [65.0-99.0]) |  |
| LGBTQ+ | 5 (8.8 [1.4-16.1]) | 5 (9.8 [1.6-18.0]) | 2 (15.4 [0.4-35.0]) |  |
| Missing | 0 | 1 | 0 |  |
| Ethnic Origin |  |  |  |  |
| White | 46 (80.7 [70.5-90.9]) | 42 (80.8 [70.1-91.5]) | 13 (100) |  |
| Minority ethnicity | 11 (19.3, 9.1-29.5) | 10 (19.2, 8.5-29.9) | 0 |  |
| Disability |  |  |  |  |
| Yes | 15 (26.8 [15.2-38.4]) | 11 (21.2 [10.1-32.3]) | 4 (30.8 [5.7-55.9]) |  |
| None | 41 (73.2 [61.6-84.8]) | 43 (82.7 [72.4-93.0]) | 9 (69.2 [44.1-94.3]) |  |
| Missing/Not stated | 1 | 0 | 0 |  |
| Mental Health comorbidity |  |  |  |  |
| Yes | 44 (77.2 [66.3-88.1]) | 37 (71.2 [58.8-83.5]) | 10 (76.9 [54.0-99.8]) |  |
| None | 13 (22.8 [11.9-33.7]) | 15 (28.8, 16.5-41.2) | 3 (23.1 [0.2-46.0]) |  |
| Employment Status |  |  |  |  |
| Employed | 20 (35.1 [24.2-49.9]) | 23 (44.2 [30.7-57.7]) | 6 (46.2 [19.1-73.3]) |  |
| Unemployed | 12 (21.1 [11.1-33.3]) | 11 (21.2 [10.1-32.3]) | 2 (15.2 [0.4-35.0]) |  |
| Long term sick or disabled | 20 (35.1 [24.2-49.9]) | 12 (23.1 [11.6-34.5]) | 2 (15.2 [0.4-35.0]) |  |
| Student | 1 (1.8 [0.1-5.4]) | 3 (5.8 [0.6-12.1]) | 2 (15.2 [0.4-35.0]) |  |
| Retired | 1 (1.8 [0.1-5.4]) | 2 (3.8 [0.1-9.1]) | 1 (7.7 [0.6-22.2]) |  |
| Other/Not Stated | 3 | 1 | 0 |  |
| Drinking Days (month) |  |  |  |  |
|  | 23.3 +/- 7.8 | 22.0 +/- 8.2 | 22.1 +/-9.7 |  |
| Daily alcohol intake |  |  |  |  |
| Daily (units) | 28 (18-39) | 26 (18-38) | 20 (15-28) |  |
| AUDIT score (median) |  |  |  |  |
|  | 32 (26-37) | 32 (28-36) | 29 (23-36) |  |
| AUDIT (category) |  |  |  |  |
| Low Risk | 0 | 0 | 0 |  |
| Increased risk | 2 (3.5 [0.1-8.3]) | 1 (1.9 [0.2-5.7]) | 1 (7.7 [0.6-22.2]) |  |
| High Risk | 3 (5.3 [0.5-11.1]) | 3 (5.8 [0.6-12.1]) | 0 |  |
| Possible dependence | 52 (91.2 [83.9-98.6]) | 48 (92.3 [85.1-99.6]) | 12 (92.3 [77.8-99.9]) |  |

Data are in mean (SD), n (% [95% CI of percentage]), median (IQR)

# Sup Table 4. Characteristics of participants who attended post randomisation baseline appointment compared to one who dropped out

|  | **Retained** |  | **Dropped out** |
| --- | --- | --- | --- |
| Age group |  |  |  |
| 18-39years | 51 (68.0 [57.4-78.6]) |  | 24 (32.0 [21.4-42.6]) |
| 40-59years | 63 (70.0 [60.5-79.5]) |  | 27 (30.0 [20.5-39.5]) |
| 60-79years | 14 (73.7 [53.9-93.5]) |  | 5 (26.3 [6.5-46.1]) |
| Gender |  |  |  |
| Male | 94 (69.1 [61.4-76.9]) |  | 42 (30.9 [23.1-38.6]) |
| Female | 34 (70.8 [58.0-83.7]) |  | 14 (29.2 [16.3-42.0]) |
| Recruitment settings |  |  |  |
| Community Drug & Alcohol Daycare Centre | 114 (67.1 [60.0-74.1]) |  | 56 (32.9 [25.9-40.0]) |
| Community Drug & Alcohol Inpatient Detoxification Unit | 7 (100) |  | 0 |
| Primary care substance use disorder clinic | 7 (100) |  | 0 |
| Ethnicity |  |  |  |
| White | 107 (68.2 [60.9-75.4]) |  | 50 (31.8 [24.6-39.1]) |
| Minority ethnicity | 21 (77.8 [62.1-93.5]) |  | 6 (22.2 [6.5-37.9]) |
| Severity of alcohol dependence |  |  |  |
| Non-dependent (SADQ 0-7) | 9 (81.8 [59.0-99.8]) |  | 2 (18.2 [0.4-41.0]) |
| Mild dependence (SADQ 8-15) | 16 (69.6 [50.8-88.4]) |  | 7 (30.4 [11.6-49.2]) |
| Moderate dependence (SADQ 16-30) | 32 (68.1 [54.8-81.4]) |  | 15 (31.9 [18.6-45.2]) |
| Severe dependence (SADQ 31-60) | 71 (68.9 [60.0-77.9]) |  | 32 (31.1 [22.1-40.0]) |
| Data are in n(% [95% CI of percentage]) |  |  |  |

# Sup Table 5. Recruitment, completion of allocated treatment and retention rates for individual services

| **Recruitment flow activity** | **Community drug & alcohol day-care centre** | **Community drug & alcohol inpatient detoxification unit** | **Primary care substance use disorder clinic** |
| --- | --- | --- | --- |
| Screened for eligibility | 365 (95.5 [92.9-97.4]) | 7 (1.8 [0.7-3.7]) | 10 (2.6 [1.2-4.7]) |
| Randomised | 170 (46.6 [41.4-51.8]) | 7 (100) | 7 (70.0 [34.7-93.3]) |
| Attended baseline visit | 114 (67.1 [59.4-74.1]) | 7 (100) | 7 (100 [59.0-100]) |
| Control group | 50 (43.9 [34.6-53.5]) | 4 (57.1 [18.4-90.1]) | 3 (42.9 [9.9-81.6]) |
| Intervention group | 64 (56.1 [46.5-65.4]) | 3 (42.9 [9.9-81.6]) | 4 (57.1 [18.4-90.1]) |
| Treatment completed | 59 (51.8 [42.2-61.2]) | 4 (57.1 [18.4-90.1]) | 3 (42.9 [9.9-81.6]) |
| Dropped out/declined | 55 (48.1 [38.8-57.8]) | 3 (42.9 [9.9-81.6]) | 4 (57.1 [18.4-90.1]) |
| Six month Follow up | 80 (70.2 [60.9-78.4]) | 4 (57.1 [18.4-90.1]) | 3 (42.9 [9.9-81.6]) |
| Died | 4 (3.5 [0.9-8.7]) | 0 | 0 |
| Data are in number (% [95% CI of percentage]) | |  |  |

# Sup Table 6. Comparison of the participants who had KLIFAD interventions versus those who did not

|  | **Transient elastography^a^** | |  | **Transient elastography +ARVS^b^** | |
| --- | --- | --- | --- | --- | --- |
|  | Yes | No |  | Yes | No |
| Age (mean) | 43.0 +/- 12.0 | 45.0 +/- 14.0 |  | 43.7 +/- 15.5 | 43.6 +/- 12.4 |
| Gender |  |  |  |  |  |
| Male | 36 (69.2, 56.7-81.8) | 14 (73.7, 53.9-93.5) |  | 9 (69.2 [44.1-94.3]) | 41 (70.7 [59.0-82.4]) |
| Female | 16 (30.8, 18.2-43.3) | 5 (26.3, 6.5-46.1) |  | 4 (30.8 [5.7-55.9]) | 17 (29.3 [17.6-41.0]) |
| Sexuality |  |  |  |  |  |
| Heterosexual | 46 (90.2, 82.0-98.4) | 17 (89.5, 75.7-99.8) |  | 11 (84.6 [65.0-99.0]) | 52 (91.2 [70.9-91.1]) |
| LGBTQ+ | 5 (9.8, 1.6-18.0) | 2 (10.5, 0.3-24.3) |  | 2 (15.4 [0.4-35.0]) | 5 (8.8 [0.8-29.1]) |
| Missing | 1 |  |  | 0 | 3 |
| Ethnic Origin |  |  |  |  |  |
| White | 42 (80.8, 70.1-91.5) | 18 (94.7, 84.7-99.9) |  | 13 (100) | 47 (81.0 [70.9-91.1]) |
| Minority ethnicity | 10 (19.2, 8.5-29.9) | 1 (5.3, 0.4-15.3) |  | 0 | 11 (19.0 [8.9-29.1]) |
| Mental Health comorbidity |  |  |  |  |  |
| Yes | 37 (71.2, 58.8-83.5) | 16 (84.2, 67.8-99.8) |  | 10 (76.9 [54.0-99.8]) | 43 (74.1 [62.9-85.4]) |
| None | 15 (28.8, 16.5-41.2) | 3 (15.8, 0.3-32.2) |  | 3 (23.1 [0.2-46.0]) | 15 (25.9 [14.6-37.1]) |
| Drinking Days (month^c^) |  |  |  |  |  |
|  | 21.9 +/- 8.2 | 24.6 +/- 6.8 |  | 21.8 +/- 9.6 | 22.8 +/- 7.8 |
| Daily alcohol intake |  |  |  |  |  |
| Daily (units) | 26 (18-38) | 20 (15-30) |  | 20 (15-28) | 28 (18-42) |
| AUDIT score (median) |  |  |  |  |  |
|  | 32 (28-36) | 31 (27-36) |  | 29 (23-36) | 32 (28-36) |
| AUDIT category |  |  |  |  |  |
| Low risk | 0 | 0 |  | 0 | 0 |
| Increasing risk (8-15) | 1 (1.9, 0.2-5.7) | 0 |  | 1 (7.7 [0.6-22.2]) | 0 |
| High risk (16-19) | 3 (5.8, 0.6-12.1) | 0 |  | 0 | 3 (5.2 [0.5-10.9]) |
| Possible dependence (≥20) | 48 (92.3, 85.1-99.6) | 19 (100) |  | 12 (92.3 [77.8-99.9]) | 55 (94.8 [89.1-99.9]) |
| Mean (SD), median (inter quartile range-IQR), number (% [95% CI of percentage]) | | | | | |
| ^a^Participants who had transient elastography and may or may not watch alcohol recovery video stories (ARVS) | | | | | |
| ^b^Participants who had transient elastography and watched ARVS vs participants who had transient elastography but did not watch ARVS | | | | | |

# Sup Table 7. Mental health comorbidity and uptake of KLIFAD intervention and engagement with services

|  | **Mental health comorbidity** | |
| --- | --- | --- |
|  | Yes | No |
| Had transient elastography |  |  |
| Yes | 37 (69.8 [57.5-82.2]0) | 15 (83.3 [66.1-99.9]) |
| No | 16 (30.2 [17.8-42.5]) | 3 (16.7 [0.5-33.9]) |
| Watched alcohol recovery video stories | |  |
| Yes | 10 (18.9[8.3-29.4]) | 3 (16.7 [0.5-33.9) |
| No | 43 (81.1 [70.6-91.7]) | 15 (83.3 [66.1-99.9]) |
| Data are number (% [95% CI of percentage]) | |  |

# Sup Table 8. Safety reporting table

|  | **Control arm (n=91)** | **Intervention arm (n=93)** |
| --- | --- | --- |
| Adverse events (AE) | 0 | 0 |
| Serious adverse events (SAE) | 0 | 0 |
| Adverse events of special interest |  |  |
| Increase in AUDIT category | 0 | 1 |
| There were four deaths reported in control arm, none of these deaths were related to trial procedures | | |

# Sup Table 9. Per-protocol analysis for change in AUDIT category at six month follow up^a^

| **AUDIT Category at baseline** | **Control group (n=57)** | **Received TE (n=52)^b^** | **Received TE and watched ARVS (n=13)^c^** |
| --- | --- | --- | --- |
| Low Risk | 0 | 0 | 0 |
| Increasing risk | 2 (3.5-0.1-8.3) | 1 (1.9, 0.2-5.7) | 1 (7.7, 0.6-22.2) |
| High Risk | 3 (5.3, 0.5-11.1) | 3 (5.8, 0.6-12.1) | 0 |
| Possible dependence | 52 (91.2, 83.9-98.6) | 48 (92.3, 85.1-99.6) | 12 (92.3, 77.8-99.9) |
| **AUDIT Category follow-up** | **Control group (n=34)** | **Received TE (n=42)** | **Received TE and watched ARVS (n=9)** |
| Low Risk | 7 (20.6, 7.0-34.2) | 17 (40.5, 25.6-55,3) | 3 (33.3, 2.5-64.1) |
| Increasing risk | 13 (38.2, 21.9-54.6) | 11 (26.2, 12.9-39.5) | 5 (55.6, 23.1-88.0) |
| High Risk | 3 (8.8, 0.7-18.4) | 5 (11.9, 2.2-21.7) | 1 (11.1, 0.9-31.6) |
| Possible dependence | 11 (32.4, 16.6-48.1) | 9 (21.4, 9.0-33.8) | 0 |
| Missing data | 23 | 10 | 4 |
| **Change in AUDIT category from baseline** | **Control group (n=34)** | **Received TE (n=42)** | **Received TE and watched ARVS (n=9)** |
| No change | 13 (38.2, 21.9-54.6) | 9 (21.4, 9.0-33.8) | 0 |
| AUDIT down in category | 21 (61.8, 45.4-78.1) | 32 (76.2, 63.3-89.1) | 9 (100) |
| AUDIT up in category | 0 | 1 (2.4, 0.2-7.0) | 0 |
| Missing data | 23 | 10 | 4 |
| **Scale of change in AUDIT category from baseline** | **Control group (n=34)** | **Received TE (n=42)** | **Received TE and watched ARVS (n=9)** |
| No change | 13 (38.2, 21.9-54.6) | 9 (21.4, 9.0-33.8) | 0 |
| AUDIT increased by one category | 0 | 1 (2.4, 0.2-7.0) | 0 |
| AUDIT reduced by one category | 5 (14.7, 2.8-26.6) | 4 (9.5, 0.6-18.4) | 1 (11.1, 0.9-31.6) |
| AUDIT reduced by two categories | 10 (29.4, 14.1-44.7) | 16 (38.1, 23.4-52.8) | 3 (33.3, 2.5-64.1) |
| AUDIT reduced by three categories | 6 (17.6, 4.8-30.5) | 12 (28.6, 14.9-42.2) | 5 (55.6, 23.1-88.0) |
| Missing data | 23 | 10 | 4 |
| Data are number (%, 95% CI of percentage), TE -transient elastography, ARVS-alcohol recovery video stories | | |  |
| ^a^This was to particularly capture participants who were no longer in services such as those who were discharge early or dropped out. | | | |
| ^b^Participant had transient elastography and received feedback based on liver stiffness measure results (LSM) may or may not watched ARVS | | | |
| ^c^Participant had transient elastography and received feedback based on liver stiffness measure (LSM) results and watched ARVS | | | |

# Sup Table 10. Comparison of participants who watched ARVS to those who did not

|  | Watched ARVs (n=13) | Did not watch ARVS (n=39) |
| --- | --- | --- |
| Age (mean) | 44+/- 16 | 42.8 +/-11.5 |
| Gender |  |  |
| Male | 9 (69.2 [44.1-94.3]) | 27 (69.2 [54.7-83.7]) |
| Female | 4 (30.8 [5.7-55.9]) | 12 (30.8 [16.3-45.3]) |
| Sexuality |  |  |
| Heterosexual | 11 (84.6 [65.0-99.0]) | 35 (92.1 [83.5-99.9]0 |
| LGBTQ+ | 2 (15.4 [0.4-35.0]) | 3 (7.9 [0.7-16.5]) |
| Missing | 0 |  |
| Ethnic Origin |  |  |
| White | 13 (100) | 29 (74.4 [60.7-88.1]) |
| Minority ethnicity | 0 | 10 (25.6 [11.9-39.3]) |
| Disability |  |  |
| Yes | 4 (30.8 [5.7-55.9]) | 5 (12.8 [2.3-23.3]) |
| None | 9 (69.2 [44.1-94.3]) | 34 (87.2 [76.7-97.7]) |
| Missing/Not stated | 0 |  |
| Mental Health comorbidity |  |  |
| Yes | 10 (76.9 [54.0-99.8]) | 27 (69.2 [54.7-83.7]) |
| None | 3 (23.1 [0.2-46.0]) | 12 (30.8 [16.3-45.3]) |
| Employment Status |  |  |
| Employed | 6 (46.2 [19.1-73.3]) | 17 (44.7 [28.9-60.5]) |
| Unemployed | 2 (15.2 [0.4-35.0]) | 9 (23.7 [10.2-37.2]) |
| Long term sick or disabled | 2 (15.2 [0.4-35.0]) | 10 (26.3 [12.2-40.3]) |
| Student | 2 (15.2 [0.4-35.0]) | 1 (2.6 [0.2-7.7]) |
| Retired | 1 (7.7 [0.6-22.2]) | 1 (2.6 [0.2-7.7]) |
| Other/Not Stated | 0 | 1 |
| Drinking Days (month) |  |  |
|  | 22.1 +/-9.7 | 21.9 +/- 7.2 |
| Daily alcohol intake |  |  |
| Daily (units) | 20 (15-28) | 28 (18-42) |
| AUDIT score (median) |  |  |
|  | 29 (23-36) | 32 (28-36) |
| AUDIT (category) |  |  |
| Low Risk | 0 | 0 |
| Increased risk | 1 (7.7 [0.6-22.2]) | 0 |
| High Risk | 0 | 3 (7.7 [0.7-16.1) |
| Possible dependence | 12 (92.3 [77.8-99.9]) | 36 (92.3 [83.9-99.9]) |

# Sup Table 11. Completion of allocated treatment program at services for patient who watched ARVS vs those who did not (per-protocol analysis)

|  |  |  |
| --- | --- | --- |
| **Per-protocol** | **Received TE and watched ARVS (n=13)** | **Received TE and did not watch ARVS (n=39)** |
| Incomplete died | 0 | 0 |
| Incomplete dropped out | 2 (15.4[4.3,42.2]) | 3 (7.7 [0.7-16.1]) |
| Incomplete declined | 3 (23.1[8.2,50.3]) | 8 (20.5 [7.8-33.2]) |
| Completed alcohol free | 2 (15.4[4.3,42.2]) | 11 (28.2 [14.1-42.3]) |
| Completed occasional alcohol user | 4 (30.8[12.7,57.6]) | 9 (23.1 [9.9-36.3]) |
| Active in services alcohol free | 0 | 5 (12.8 [2.3-23.3]) |
| Active in service occasional alcohol user | 2 (15.4[4.3,42.2]) | 3 (7.7 [0.7-16.1]) |
| Active in service increased alcohol intake | 0 | 0 |
| Data are number (% [95% CI]), TE-transient elastography, ARVS-alcohol recovery video stories | |  |

# Sup Table 12. Change in self-reported measures of alcohol at six-month follow-up for participants who watched ARVS vs those who did not

| Per-protocol | **Received TE and watched ARVS (n=9)** | **Received TE and did not watch ARVS (n=39** |
| --- | --- | --- |
| Change in drinking days (mean) | -6.7 +/- 10.2 | -13.4 +/-10.5 |
| Change in daily units (median) | -17.0 (-27.0, -11.1) | -25.0 (-37.0, -12.0) |
| Change in AUDIT (median) | -21.0 (-24.0, -15.0) | -19 (--28.5, -11.5) |
| Data are given in mean (SD), median (interquartile range -IQR) TE -transient elastography, ARVS-alcohol recovery video stories | | |

# Sup Table 13. Change in AUDIT for patient who watched ARVS vs those who did not (per-protocol analysis)

| **AUDIT Category at baseline** | **Received TE and watched ARVS (n=13)** | **Received TE and did not watch ARVS (n=39)** |
| --- | --- | --- |
| Low Risk | 0 | 0 |
| Increasing risk | 1 (7.7, 0.6-22.2) | 0 |
| High Risk | 0 | 3 (7.7, 0.7-16.1) |
| Possible dependence | 12 (92.3, 77.8-99.9) | 36 (92.3, 83.9-99.9) |
| **AUDIT Category follow-up** | **Received TE and watched ARVS (n=9)** | **Received TE and did not watch ARVS (n=39)** |
| Low Risk | 3 (33.3, 2.5-64.1) | 14 (42.4, 25.6-59.3) |
| Increasing risk | 5 (55.6, 23.1-88.0) | 6 (18.2, 5.0-31.3) |
| High Risk | 1 (11.1, 0.9-31.6) | 4 (12.1, 1.0-23.3) |
| Possible dependence | 0 | 9 (27.3, 12.1-42.5) |
| Missing data | 4 | 6 |
| **Change in AUDIT category from baseline** | **Received TE and watched ARVS (n=9)** | **Received TE and did not watch ARVS (n=39)** |
| No change | 0 | 9 (27.3, 12.1-42.5) |
| AUDIT down in category | 9 (100) | 23 (69.7, 54.0-85.4) |
| AUDIT up in category | 0 | 1 (3.0, 0.2-8.9) |
| Missing data | 4 | 6 |
| **Scale of change in AUDIT category from baseline** | **Received TE and watched ARVS (n=9)** | **Received TE and did not watch ARVS (n=39)** |
| No change | 0 | 9 (27.3, 12.1-42.5) |
| AUDIT increased by one category | 0 | 1 (3.0, 0.2-8.9) |
| AUDIT reduced by one category | 1 (11.1, 0.9-31.6) | 3 (9.1, 0.7-18.9) |
| AUDIT reduced by two categories | 3 (33.3, 2.5-64.1) | 7 (21.2, 7.3-35.2) |
| AUDIT reduced by three categories | 5 (55.6, 23.1-88.0) | 13 (39.4, 22.7-56.1) |
| Missing data | 4 | 6 |
| Data are number (%, 95% CI of percentage), TE -transient elastography, ARVS-alcohol recovery video stories | | |

# Sup Table 14. Median (IQR) for a few outcomes by settings (treatment site)

| **Outcome variable** | **Setting** | | |
| --- | --- | --- | --- |
|  | **Community drug & alcohol inpatient detoxification unit (n=7)** | **Community drug & alcohol day-care centre (n=114)** | **Primary care substance use disorder clinic (n=7)** |
| Drinking days per month | 28 (28-28) | 28 (20-28) | 28 (28-28) |
| Daily alcohol intake units | 28 (26-38) | 26 (18-37) | 14 (6-25) |
| AUDIT score | 36 (34-37)* | 32 (28-36) | 21 (12-34) |
| SADQ Score | 35 (35-49) | 32 (21-41) | 16 (0-39) |
| *based on n=4 |  |  |  |

# Sup Table 15. Estimated ICC by trial arm based on baseline values of few outcome variables

| **Outcome variable** | **Trial arm** | |
| --- | --- | --- |
|  | **Control (n=71)** | **Intervention (n=57)** |
| Drinking days per month | 0.069 | 0.118 |
| Daily alcohol intake units | 0 | 0.09 |
| AUDIT score | 0.346 | 0.362 |
| SADQ score | 0.2 | 0.1 |
